# Enhanced PKCδ and ERK Signaling Mediate Cell Migration of Retinal Pigment Epithelial Cells Synergistically Induced by HGF and EGF

**DOI:** 10.1371/journal.pone.0044937

**Published:** 2012-09-20

**Authors:** Yu Jung Chen, Rong Kung Tsai, Wen Chen Wu, Ming Shan He, Ying-Hsien Kao, Wen Sheng Wu

**Affiliations:** 1 Department of Laboratory Medicine and Biotechnology, College of Medicine, Tzu Chi University, Hualien, Taiwan; 2 Department of Ophthalmology, Buddhist Tzu Chi General Hospital, Hualien, Taiwan; 3 Department of Ophthalmology and Visual Science, Tzu Chi University, Hualien, Taiwan; 4 Department of Ophthalmology, Kaohsiung Medical University, Kaohsiung, Taiwan; 5 Department of Medical Research, E-DA Hospital, I-Shou University College, Kaohsiung, Taiwan; Chang Gung University, Taiwan

## Abstract

Proliferative vitreoretinopathy (PVR) and proliferative diabetic retinopathy (PDR) are characterized by the development of epi-retinal membranes which may exert a tractional force on retina. A lot of inflammatory growth factors may disturb the local ocular cells such as retinal pigment epithelial (RPE) cells, causing them to migrate and proliferate in the vitreous cavity and ultimately forming the PVR membrane. In this study, the signal pathways mediating cell migration of RPE induced by growth factors were investigated. Hepatocyte growth factor (HGF), epidermal growth factor (EGF) or heparin-binding epidermal growth factor (HB-EGF) induced a greater extent of migration of RPE50 and ARPE19 cells, compared with other growth factors. According to inhibitor studies, migration of RPE cells induced by each growth factor was mediated by protein kinase C (PKC) and ERK (MAPK). Moreover, HGF coupled with EGF or HB-EGF had synergistic effects on cell migration and enhanced activation of PKC and ERK, which were attributed to cross activation of growth factor receptors by heterogeneous ligands. Furthermore, using the shRNA technique, PKCδ was found to be the most important PKC isozyme involved. Finally, vitreous fluids from PVR and PDR patients with high concentration of HGF may induce RPE cell migration in PKCδ- and ERK- dependent manner. In conclusion, migration of RPE cells can be synergistically induced by HGF coupled with HB-EGF or EGF, which were mediated by enhanced PKCδ activation and ERK phosphorylation.

## Introduction

Proliferative vitreoretinopathy (PVR) is the most common cause of surgical failure for rhegmatogenous retinal detachment [1], [2] which, along with proliferative diabetic retinopathy (PDR), is one of the most important causes of blindness. PVR and PDR are characterized by the development of fibrous membranes within the vitreous cavity and on retinal surfaces (an epi-retinal membrane) which may exert a tractional force on retina that reopens otherwise successfully treated retinal breaks [3]–[5]. The pathological process of PVR begins with retinal breaks and the subsequent inflammation [5]–[8], during which a lot of growth factors such as platelet derived growth factor (PDGF) [9], transforming growth factor β (TGFβ) [10], heparin-binding epidermal growth factor (HB-EGF) [11], hepatocyte growth factor (HGF) [12], [13] and epidermal growth factor (EGF) [14] may be released into the vitreous cavity. These factors may disturb the local ocular cells such as retinal pigment epithelial cells (RPE) and glial cells to migrate and proliferate in the vitreous cavity, forming the PVR membrane [5], [15]. RPE cells are considered as key element in the development of PVR [15] and present in almost 100% of epiretinal membranes, with diverse morphologic characteristics [16]. In the past decades, it was well established that HGF [17], [18], EGF [17], [19], [20], PDGF [21], [22] and TGFβ [23], [24] may trigger a variety of PVR-related phenotypical changes of RPE, including proliferation and migration.

It is established that extracellular signal-regulated kinase (ERK) of the mitogen activated protein kinase (MAPK) family is essential for mediating migration of RPE induced by EGF [17], [19], PDGF [25] and HGF [17], [26], [27]. Frequently, phosphoinositide 3-kinase (PI3K)-AKT pathway is activated along with ERK for mediating RPE migration induced by PDGF [22], EGF and HGF [17]. Protein kinase C (PKC), known to be essential for retinal pathophysiology, is required for a lot of cellular processes of RPE including proliferation [28] and migration [29]. However, whether it is responsible for the PVR-related cellular changes of RPE has not been reported.

One intriguing issue is whether the inflammatory growth factors that elevate concomitantly within the ocular microenvironment trigger the PVR-related phenotypical changes in a synergistic manner. Early studies demonstrated that several growth factors cooperated to enhance the molecular and cellular effects of RPE [30]–[32]. Importantly, a recent report demonstrated that HGF and HB-EGF in combination may enhance RPE cell migration *via* signal cross talk [17].

In this study, the signal pathways mediating the PVR-related changes of RPE induced by HGF, HGF, HB-EGF, TGFβ and PDGF were investigated. Specifically, we found HGF coupled with EGF or HB-EGF induced migration of RPE in a synergistic manner, *via* enhancement of PKCδ and ERK.

## Materials and Methods

### Human RPE cell lines

A primary culture of human RPE50 cells was provided by the Tissue Culture Center, New York Eye and Ear Infirmary. This cell line was isolated from an anonymous donor sample not referable to any patient [33]. RPE50 have been used for studying the effects of oxidative stress on ion channels [34], cell cycle and gene expression [35] in human RPE cells. Human ARPE19 was purchased from the Bioresource Collection and Research Center (BCRC, Hsinchu, Taiwan). ARPE-19 cells have been used in a lot of studies such as cross talk between c-Met and epidermal growth factor receptor during RPE wound healing [17]. Both cell lines were maintained in a 1∶1 mixture of Dulbecco's modified Eagle's medium (DMEM) and F12 medium.

### Chemicals and Antibodies

Bisindolymaleimide and AG1478 were purchased from Calbiochem (Darmstadt, Germany). Worthmannin was from Sigma (St. Louis, MO, USA). Rottlerin, Go6976, Ro32-0432 and HBDDE were from Enzo Life Science (Plymouth meeting, PA, USA). HGF and EGF were from Peprotech (Rocky Hill, NJ, USA). HB-EGF was from R&D (Minneapolis, MN, USA), and JNJ38877605 from Selleck (Houston, TX, USA). Antibodies against p-ERK, ERK and PKCα, β, γ, δ were from Santa Cruz (California, CA, USA).

### Wound Healing Migration Assay

Briefly, RPE cells were seeded in 24-well plate accompanied by a wound healing culture insert and grown into confluence, followed by serum starvation for 48 h. After removal of culture insert, the cells were treated under appropriate conditions for 18 h. Motility of the cells were quantitated by directly counting the cells migrated into the blanking area under phase contrast microscope at 200-fold magnification.

### Fractionation of Cellular Extract for PKC assay

Briefly, the cells were suspended in hypotonic buffer and incubated at 4°C for 30 min followed by homogenization and centrifugation at 2500 rpm for 3 min. The supernatants were then subjected to ultracentrifugation at 25000 rpm for 1 hr, and the resulting supernatants were obtained as the cytosolic fraction. The pellets were then dissolved in lysis buffer containing 1% Triton X-100. Following a second centrifugation at 25000 rpm for 1 hr, the supernatants were collected and used as the membrane fraction.

### PKC Activity Assay

PKC activity in membrane fraction isolated as described above was determined using PKC Elisa kit (Enzo Life Science, Plymouth Meeting, PA, USA) according to manufacture's protocol.

### Western Blot

Western blot was performed according to the standard procedure. For quantification, the intensity of each specific band was estimated with gel digitizing software UN-SCAN-IT gel version 5.1.

### siRNA Technology for Depletion of PKC Isozymes

Lentiviral plasmids each encoding shRNAs targeting different regions of indicated mRNA were obtained from RNAi Core Laboratory in Academia Sinica, Taiwan. Cells were transfected with shRNAs using Effectene transfection reagent (GIBOCO, Gaithersburg, USA) according to the manufacturer's protocol.

### Vitreous sample and detection of growth factor

Vitreous fluids were collected from PVR and PDR retinal detachment (RD) patients who underwent vitrectomy as part of the therapeutic regimen at Kaohsiung Medical University Hospital, Kaohsiung, Taiwan. The collection protocol was approved by the Institutional Review Board of Kaohsiung Medical University (Protocol No. KMUH-IRB-970387). All subjects were given their full written informed consent. Assays of HGF, EGF and HB-EGF in vitreous fluids were performed using Elisa kit for each growth factor (R&D., Minneapolis, MN, USA) according to manufacture's protocol.

### Statistical Analysis

Pair Student's *t* test was done to analyze the statistical significance of between-group differences. All quantitative studies were performed at least in triplicate, with the results expressed as means ± SD as appropriate. Differences were considered to be significant at P<0.05.

## Results

### Effects of combined growth factorson migration of RPE cells

**Figure 1:**
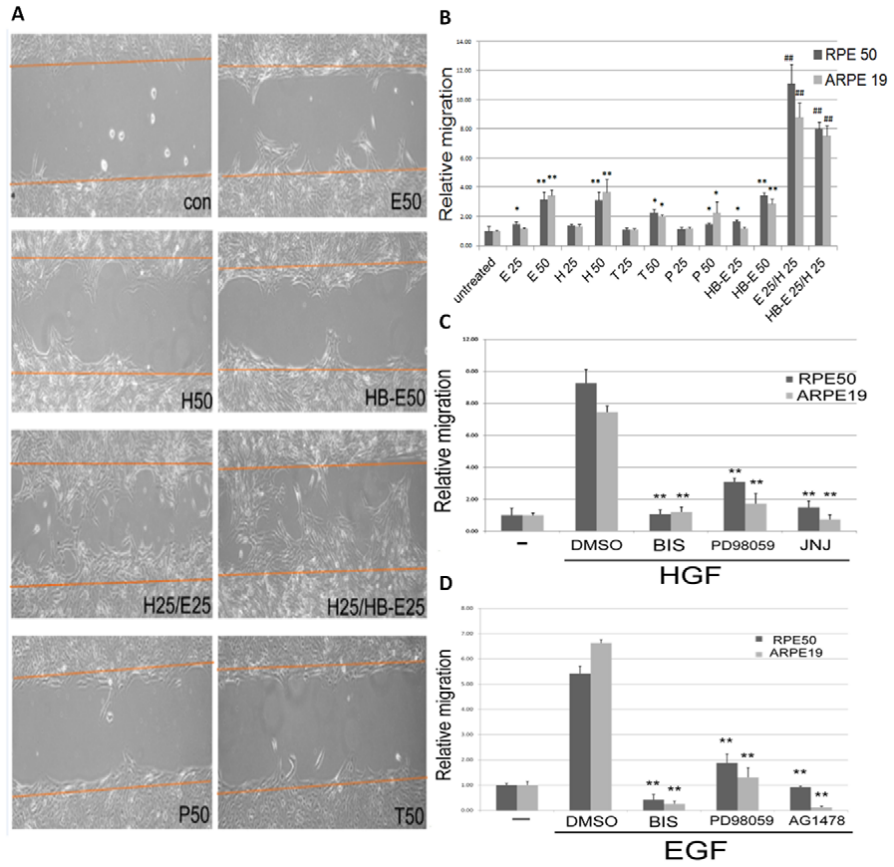
Growth factors induced cell migration of ARPE19 and RPE50. (A) ARPE 19 was cultivated with a wound healing culture insert and serum-starved for 48 h. After removal of culture insert (0 h), cells were treated with 50 nM of HGF (H50), EGF (E50), TGFβ1 (T50) or PDGF (P50), 50 ng/ml HB-EGF (HB-E50), 25 nM HGF coupled with 25 nM EGF (H25/E25) or 25 nM HGF coupled with 25 ng/ml EGF (H25/HB-E25) in serum free medium for 18 h and photographed. The cells migrated into the blanking area between the indicated orange lines representing the boundary between the blanking area and cell culture at 0 h. The results were representatives of four reproducible experiments. (B) Quantitative analysis of relative migration of ARPE19 (gray) and RPE 50 (black) treated with growth factors as indicated in (A) and supplemental **Figure 1**, respectively. The number of cells migrated into the wound area were counted under phase contrast microscope (at 200-fold magnification). For each treatment, total cell numbers in four microscopic fields were counted. Relative migration of the cells were calculated as the ratio of cell numbers of each treatment *vs.* that of untreated cells, taken the ratio of untreated cells as 1.0. The results were averages of three experiments with coefficient of variation (C.V.) of 5.5%. (*) and (**) represent statistical significance (P<0.05 and P<0.005, respectively) for comparison of the growth factor-treated with untreated group. (^##^) represent statistical significance (P<0.005) for comparison of the combined growth factors *vs.* single growth factors group as described in the text. (C) and (D) RPE 50 (black) and ARPE19 (gray) were untreated (-), treated with 50 nM HGF (C) and EGF (D), or HGF and EGF coupled with DMSO (as solvent control) or various inhibitors as indicated. Wound healing assay were performed and relative migrations were quantitated as described in (B). JNJ in (C): JNJ38877605. The results were averages of 3 experiments with coefficient of variation (C.V.) of 5.5%. (**) represent statistical significance (P<0.005) for comparison of the inhibitor- *vs.* DMSO-treated group.

Initially, the dose-dependent effects of various growth factors on cell migration of both ARPE19 and RPE50 were investigated using a wound healing method. As demonstrated in **Figure 1A**, 50 nM HGF or EGF, or 50 ng/ml HB-EGF may significantly promote migration of ARPE19 whereas the effects of 50 nM TGFβ1 or PDGF were less prominent. Also, cell migration of both RPE cells can be slightly induced by 25 nM HGF or EGF, or 25 ng/ml HB-EGF but not by 25 nM TGFβ1 or PDGF (data not shown). The similar growth factor-induced migratory effects were also observed in RPE50 (**Figure S1**). Quantitative analysis revealed that 50 nM HGF or EGF, or 50 ng/ml HB-EGF induced cell migration of both RPE cells to an extent (3.1–3.8 fold) greater than TGFβ1 or PDGF did (1.5–2.2 fold) (**Figure 1 B**). We further investigated whether migration of RPE could be synergistically induced by these growth factors. Interestingly, combined treatments of (25 nM HGF/25 nM EGF) and (25 nM HGF/25 ng/ml HB-EGF) induced higher motility of ARPE19 (**Figure 1A**) and RPE50 (**Figure S1**) as compared with that induced by each single growth factor at 2-fold concentration (*i.e.*50 nM HGF or EGF, or 50 ng/ml HB-EGF ). Quantitative analysis (**Figure 1B**) revealed that the enhanced effects of (25 nM HGF/25 nM EGF) and (25 nM HGF/25 ng/ml HB-EGF) on cell migration of both RPE cells were 2.1–2.5 and 4.4–5.2 fold compared with those of each single growth factor at 2-fold and same (*i.e.*25 nM HGF or EGF, or 25 ng/ml HB-EGF) concentration, respectively. In contrast, combinations of EGF/TGFβ, HGF/PDGF and HGF/TGFβ didn't induce such a synergistic effect (data not shown). In addition, cell proliferation of both RPE cells was not affected by the aforementioned growth factors within 48 h using 3-(4,5-cimethylthiazol-2-yl)-2,5-diphenyl tetrazolium bromide (MTT) method (data not shown).

### PKC and ERK mediated Growth factor-induced RPE Migration

We further investigated the signal mechanisms for cell migration triggered by the aforementioned growth factors. Initially, various inhibitors against critical signal components for RPE including PKC [29], ERK (MAPK) [17], [19], [25]–[27], AKT [17], [22] were employed. As demonstrated in **Figure 1C and D**, bisindolymaleimide (BIS), the inhibitors of PKC, can totally suppress migration of RPE50 and ARPE19 induced by HGF (**Figure 1C**) and EGF (**Figure 1D**), whereas PD98059, the inhibitor of MEK (which is the upstream kinase of ERK) exhibited 70%–80% migration suppression. As positive controls, JNJ38877605 and AG1478, the inhibitors of c-met (the receptor of HGF) and EGFR (the receptor of both EGF and HB-EGF) abolished migration of both types of RPE cells induced by HGF and EGF, respectively. In addition, HB-EGF-induced cell migration of RPE50 was also abolished by AG1478, BIS and PD98059 (**Figure S2**). Worthmannin, the AKT inhibitor, didn't prevent cell migration of RPE induced by the growth factors (data not shown), suggesting AKT was not involved. In summary, HGF, HB-EGF or EGF may trigger migration of RPE which was mediated by PKC and ERK.

### Induction of PKC/ERK Signal cascade by HB-EGF, EGF and HGF

**Figure 2:**
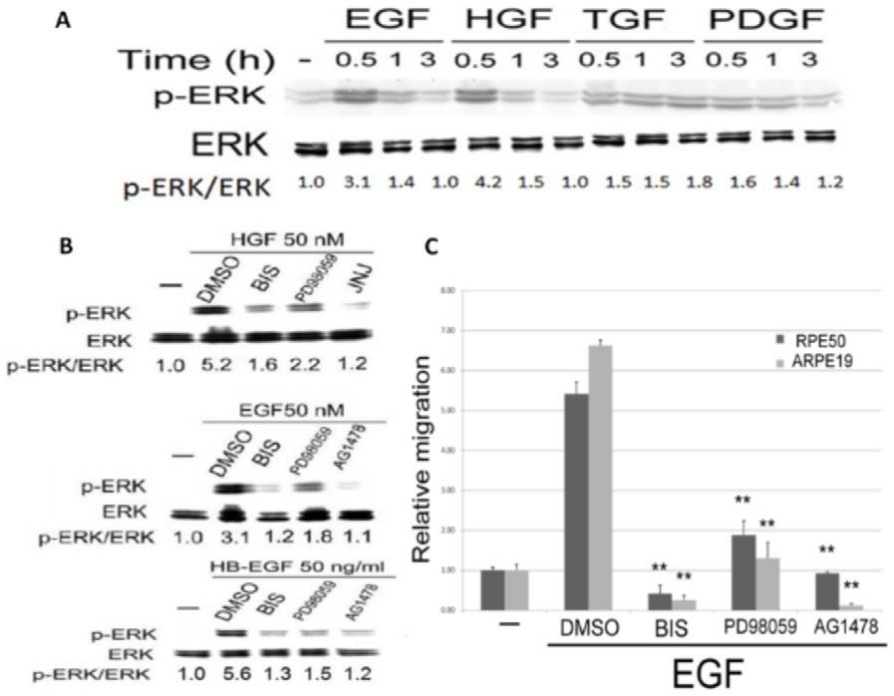
Growth factors induced ERK phosphorylation in RPE50 and ARPE19. (A) RPE50 cells were untreated (-), treated with 50 nM of HGF, EGF, TGFβ and PDGF for the time indicated. (B) RPE50 cells were untreated (-), treated with 50 nM of HGF or EGF or 50 ng/ml HB-EGF coupled with DMSO or various inhibitors as indicated for 0.5 h. (C) RPE50 (upper panel) and ARPE 19 (lower panel) cells were untreated (a and b), treated with 50 nM of HGF (H50) (a and b) and EGF (E50) (a), 50 ng/ml HB-EGF (HB-E 50) (b), 25 nM of EGF and HGF in combination (E25/H25) (a) or 25 ng/ml HB-EGF and 25 nM HGF in combination (HB-E25/H25) (b) for 0.5 h. Western blot of p-ERK were performed using ERK as internal control. The relative ratios of the intensity for p-ERK/ERK (indicated below each lane) were calculated, taking the ratio of the untreated group as 1.0. The results were averages of 2 experiments with coefficient of variation (C.V.) of 6.0–7.0%.

**Figure 3:**
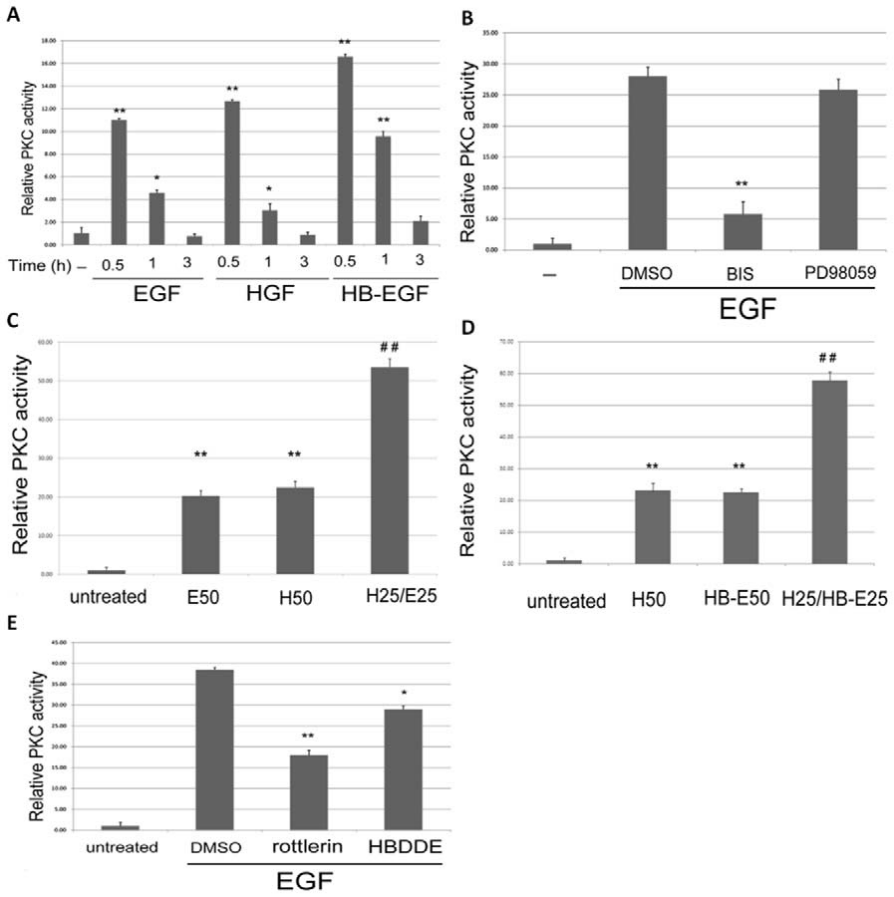
Growth factors induced PKC activation in RPE50 and ARPE19. (A) RPE50 cells were untreated (-), treated with 50 nM of HGF or EGF, or 50 ng/ml HB-EGF for the time indicated. (B) and (E) RPE50 cells were untreated (-), treated with 50 nM EGF or EGF coupled with DMSO or various inhibitor as indicated for 0.5 h. (C) and (D) RPE50 cells were untreated, treated with 50 nM of HGF (H50) or EGF (E50), 50 ng/ml HB-EGF (HB-E50), 25 nM of EGF and HGF in combination (E25/H25) or 25 ng/ml HB-EGF and 25 nM HGF in combination (HB-E25/H25) for 0.5 h. PKC activity assay were performed. Relative PKC activity was obtained as the ratio of calculated PKC activity of each treatment *vs.* that of untreated cell, taking the ratio of untreated cell as 1.0. The results were averages of 3 experiments with coefficient of variation (C.V.) of 5.5%. In (A), (*) and (**) represent statistical significance (P<0.05 and P<0.005, respectively) for comparison of the growth factor-treated *vs.* untreated group. In (B) and (E), (*) and (**) represent statistical significance (P<0.05 and P<0.005, respectively) for comparison of the inhibitor- *vs.* DMSO-treated group. In (C) and (D), (**) represent statistical significance (P<0.005) for comparison of the single growth factor-treated *vs.* untreated group. (^##^) represent statistical significance (P<0.005) for comparison of the combined growth factors-treated *vs.* single growth factor-treated group as described in the text.

Whether PKC and ERK can be activated by the aforementioned growth factors were further examined. As demonstrated in **Figure 2A**, phosphorylation of ERK can be greatly induced to 3.1–4.2 folds by 50 nM EGF or HGF within 0.5 h, and thereafter declined to basal level within 3 h in RPE50 cells. In contrast, phosphorylation of ERK can only be slightly induced by 50 nM PDGF or TGFβ (1.5–1.8 fold) within 0.5 to 3.0 h. Also, 50 ng/ml HB-EGF induced ERK phosphorylation in RPE50 with a time course similar to that of EGF and HGF did (**Figure S3**). Similar growth factor-induced ERK phosphorylation was also observed in ARPE19 (data not shown). In summary, HB-EGF, EGF and HGF induced transient but robust phosphorylation of ERK at 0.5 h after treatment, which is different from the sustained but weak phosphorylation of ERK induced by PDGF and TGFβ. For analyzing the activation of PKC, a nonradioactive PKC assay was performed. As demonstrated in **Figure 3A**, EGF (50 nM), HGF (50 nM) and HB-EGF (50 ng/ml) elevated PKC activity in RPE50 cells to 11, 12.5 and 16.5-fold, respectively, which dramatically declined at 1 h and totally abolished at 3 h. Since the time course for PKC activation was similar with that for ERK activation, it is very probable that both kinases are within the same signal cascade. Indeed, HGF-induced phosphorylation of ERK at 0.5 h can be reduced by BIS, PD98059 and JNJ38877605 by 70, 58 and 77% respectively (Fig. 2B upper panel). Similarly, phosphorylation of ERK induced by EGF and HB-EGF at 0.5 h can be suppressed by BIS, PD98059 and AG1478 by 70–90, 65–90 and 95–100%, respectively (**Figure 2B**, middle and lower panel). On the contrary, activation of PKC induced by all three growth factors at 0.5 h was not prevented by PD98059 in RPE50 (**Figure 3B** and data not shown). Collectively, these results strongly suggested that PKC acts upstream of ERK in the signal pathway mediating migration of RPE induced by these growth factors.

### Effects of Combined Growth factors on Signal transduction and RPE cell Migration

**Figure 4:**
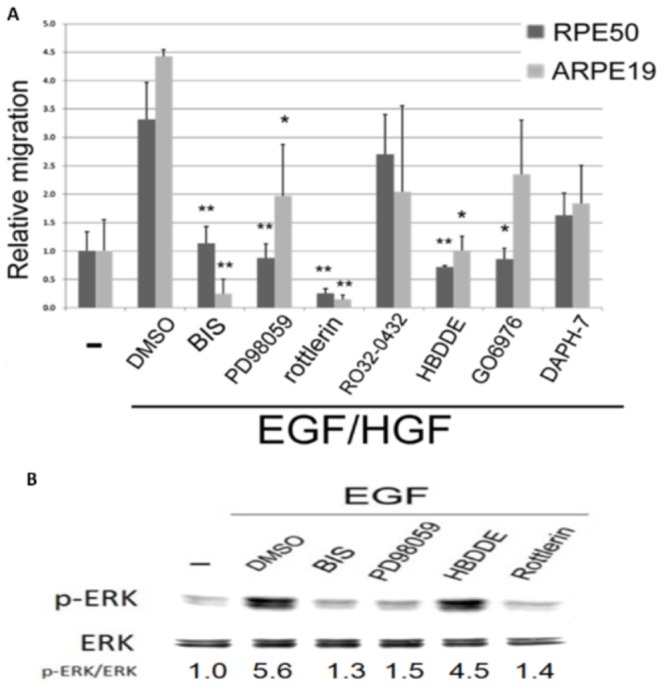
Prevention of growth factors-induced cell migration and ERK phosphorylation of RPE by inhibitors of PKC isozymes. RPE50 (black) (A, B) and ARPE 19 (gray) cells (A) were cultivated with a wound healing culture insert and depleted of serum for 24 h. After removing the culture insert, cells were untreated (-), treated with 25 nM HGF/EGF in combination (A) or 50 nM EGF (B), or those coupled with DMSO, PD98059 or various PKC isozyme inhibitors as indicated for 18 h. Wound healing assay were performed and quantitated as described in Fig. 1B. (*) and (**) represent statistical significance (P<0.05 and P<0.005, respectively) for comparison of the inhibitors-treated *vs.* DMSO-treated group. The results were averages of 5 experiments with coefficient of variation (C.V.) of 5.5%. (C) RPE50 cells were untreated (-), treated with 50 nM of EGF or EGF coupled with DMSO or various inhibitors as indicated for 0.5 h. Western blot of p-ERK were performed using ERK as internal control. The relative ratios of the intensity for p-ERK/ERK (indicated below each lane) were calculated, taking the ratio of the untreated group as 1.0. The results were averages of 2 experiments with coefficient of variation (C.V.) of 6.0–7.0%.

Whether the activations of PKC and ERK were enhanced by simultaneous action of the growth factors was further investigated. Interestingly, combinations of (25 nM HGF/25 nM EGF) and (25 nM HGF/25 ng/ml HB-EGF), induced a greater extent of ERK phosphorylation in RPE50 and ARPE19 by 2.5–2.8 and 1.5–2.2 fold, respectively, compared with each single growth factor at 2-fold concentration (**Figure 2C**). Consistently, combination of (25 nM HGF/25 nM EGF) (**Figure 3C**) or (25 nM HGF/25 ng/ml HB-EGF) (**Figure 3D**) induced greater PKC activation in RPE50 by 2.5–3.1-fold, compared with each growth factor at 2-fold concentration. On the other hand, cell migrations of RPE50 and ARPE19 induced by combination of (25 nM HGF/25 nM EGF) were effectively abolished by BIS or PD98059, but not DMSO, by 75–100% (**Figure 4A**). Together, these results suggested that enhanced activations of both ERK and PKC were responsible for synergistic effects on RPE cell migration induced by HGF coupled with EGF or HB-EGF.

### PKCδ was specifically required for Synergistic cell Migration induced by Growth factors

**Figure 5:**
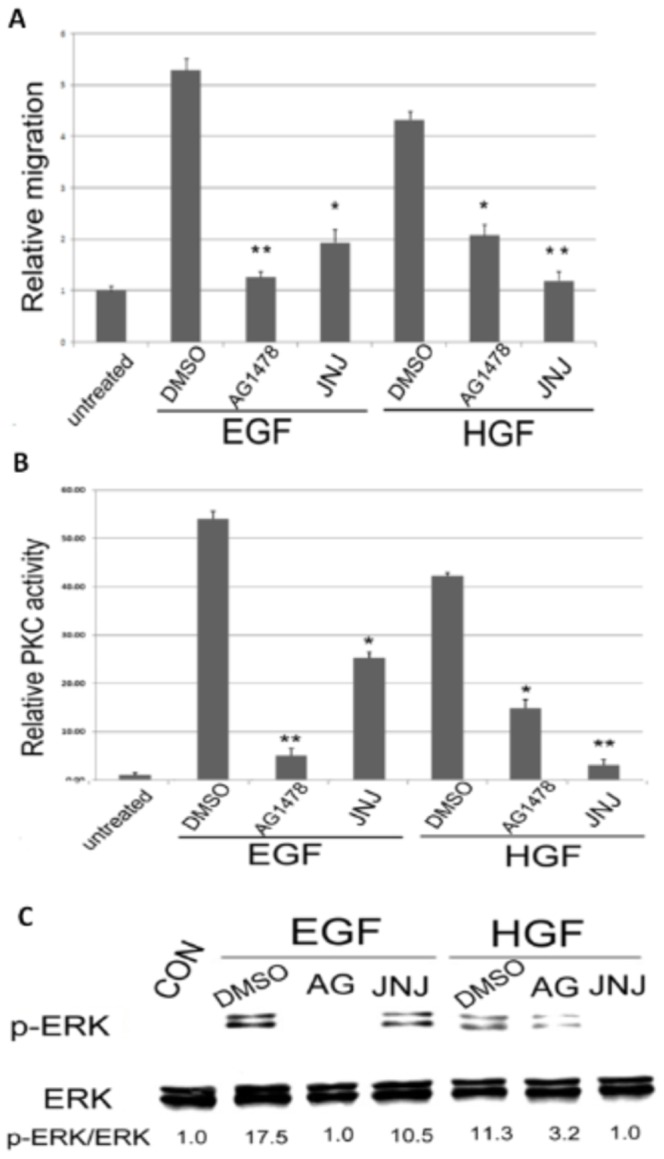
Prevention of growth factors-induced cell migration and ERK phosphorylation of RPE50 and ARPE19 by depletion of PKC isozymes. (A) RPE50 (black) and ARPE 19 (gray) cells were cultivated with a wound healing culture insert for 24 h, transfected with shRNA of GFP or PKC α, β or δ for 36 h followed by treatment with 50 nM EGF in serum free medium for 18 h. Wound healing assay were performed and quantitated as in **Figure 1B**. (*) and (**) represent statistical significance (P<0.05 and P<0.005, respectively) for comparison of the PKC isozyme shRNA *vs.* GFP shRNA group. (B) and (C) RPE50 was transfected with shRNA of GFP or PKCα, β or δ for 36 h followed by treatment with 50 nM EGF for 0.5 h (B) or none (C). Western blot of p-ERK (B) and PKCα, β or δ(C) were performed using ERK as internal control. The relative ratios of the intensity for p-ERK/ERK (B) and PKC isozyme/ERK (C) were calculated, taking the ratio of the untreated group as 1.0. The results were averages of 2 experiments with coefficient of variation of 6.0–7.0%.

We further investigate which PKC isozymes were involved in mediating ERK phosphorylation and cell migration of RPE cells. There are more than 12 PKC isozymes [36], [37] among which PKCα, PKC βII, PKC δ and PKCε were known to be responsible for pathophysiological effects of RPE [28]. Various inhibitors were employed for identification of the PKC isozymes involved. As demonstrated in **Figure 4A**, 2 µM Rottlerin (for PKCδ, IC50 = 2–6 µM), 5 nM Go6976 (for PKCα, β and γ, IC50 = 2–10 nM), 28 nM Ro32-0432 (for PKCα and βI, IC50 = 9 nM, 28 nM), 500 nM DAPH-7, (for PKCβII and βI, IC50 = 410 nM, 3.8 µM) 50 µM HBDDE (for PKCα, IC50 = 43 µM) or 5 µM BIS (for PKCα, δ, ζ, η and ηε, IC50 = 1–5 µM) effectively suppressed cell migration of RPE50 and ARPE19 induced by 25 nM HGF/25 nM EGF. Specifically, Rottlerin, the inhibitor of PKCδ, abolished cell migration of both RPE50 and ARPE19 induced by HGF/EGF to a level even lower than the basal migratory activity. Also, HBDDE, the inhibitor of PKCα, totally suppressed the HGF/EGF-induced cell migration of both RPE cells (**Figure 4A**). In the single growth factor-treated group, Rottlerin and BIS almost prevented EGF (50 nM) -induced cell migration of RPE50 by 95%, whereas HBDDE exhibited only 42% suppressive effect (**Figure 4 B**). Consistently, Rottlerin suppress EGF-induced ERK phosphorylation (by 88%) in RPE50 as effectively as BIS (by 93%) whereas HBDDE exerted much less suppressive effect (by 17%) (**Figure 4C**). In addition, PKC activity induced by EGF was suppressed by Rottlerin and HBDDE by 52% and 35%, respectively, in RPE50 (**Figure 3E**). To further confirm the PKC isozymes involved, cells were transfected with different shRNA fragments of various PKC isozymes for 36 h prior to growth factor treatment. As shown in **Figure 5A**, EGF-induced cell migration of ARPE19 and RPE50, which were transfected separately with shRNA (fragment E, F, G or H) of PKCδ, decreased by 50–100% compared with that transfected with control shRNA of green fluorescent protein (GFP) or none (MOCK). In contrast, shRNAs of both PKCα (fragment 90 or 92) and PKCβ (fragment A2 or O) did not prevent EGF-induced cell migration of RPE50 at all, and prevented EGF-induced cell migration of ARPE19 in a much less extent than shRNA of PKC δ. Moreover, shRNA of PKCδ (but not PKCα or PKCβ) dramatically suppressed ERK phosphorylation in RPE50 induced by the combination of (25 nM HGF/25 nM EGF) (**Figure 5B**). Western blots (**Figure 5C**) demonstrated that PKCα, βII and δ were effectively depleted after transfection with each own shRNA for 36 h. Taken together, PKCδ is the most important PKC isozyme mediating ERK phosphorylation and cell migration of RPE induced by HGF and/or EGF. This was further supported by that transient over-expression of PKCδ (**Figure S4A**) enhanced EGF-induced cell migration (**Figure S4B**) and ERK phosphorylation (**Figure S4C** ) by 48 and 57%, respectively.

### Cross activations of the growth factor receptors were implicated in signaling enhancement

**Figure 6:**
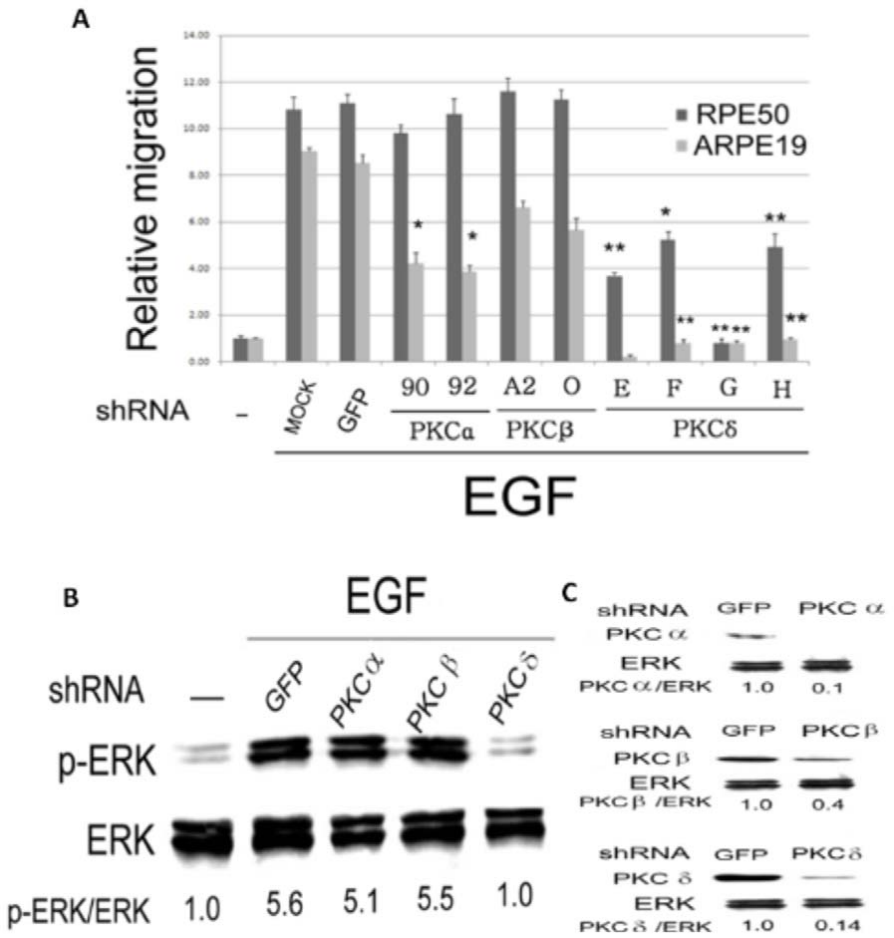
Inhibitors of the of EGF and HGF receptors prevented migration and ERK phosphorylation reciprocally induced by HGF and EGF. RPE50 cells were untreated, treated with 50 nM of HGF or EGF coupled with the indicated inhibitors (AG: AG1478, JNJ: JNJ38877605) or DMSO (as solvent control) for 24 h (A) or 30 min (B and C). Wound healing cell migration assay (A), PKC assay (B) and Western blot of p-ERK (C) were performed. In (A) and (B), (*) and (**) represent statistical significance (P<0.05 and P<0.005, respectively) for comparison of the inhibitors-treated *vs.* DMSO-treated group. Western blot of ERK served as internal control for (C). The relative ratios of the intensity for p-ERK/ERK (indicated below each lane) were calculated, taking the ratio of the untreated group as 1.0. The results were averages of 2 experiments with coefficient of variation (C.V.) of 6.0–7.0%.

Previous study [17] suggested HGF and HB-EGF may collaborate to enhance cell migration via signaling cross talk, probably due to the cross activation of each growth factor receptor by heterogeneous ligands. As demonstrated in **Figure 6A**, JNJ38877605, the inhibitor of HGF/c-met partially suppress cell migration of RPE50 induced by EGF by 45%, compared with the 76% inhibitory effect exerted by AG1478. On the other hand, AG1478 partially suppress HGF-induced cell migration of RPE by 52%, compared with the 72% inhibitory effect exerted by JNJ38877605. Consistently, EGF-induced PKC activation (**Figure 6B**) and ERK phosphorylation (**Figure 6C**) of RPE50 was partially suppressed ( by 43–50%) by JNJ38877605, whereas those induced by HGF can be suppressed (by 65%–72%) by AG1478. Collectively, these results suggested that the receptors of EGF and HGF were reciprocally activated by HGF and EGF, respectively, contributing to enhancement of down stream PKC/ERK signaling.

### Vitreous samples of PVR and PDR induce cell migration of RPE via ERK/PKC pathway

**Figure 7:**
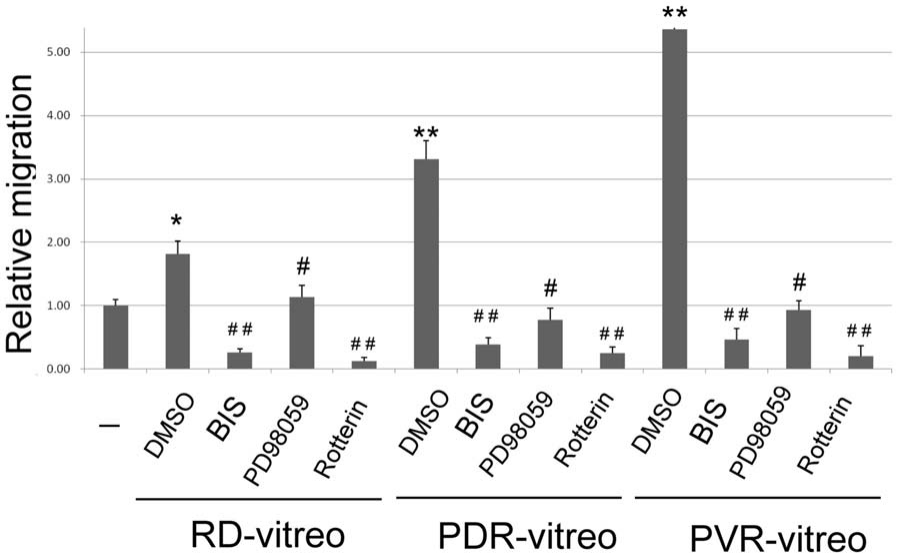
Prevention of cell migration of RPE50 induced by vitreous fluid by inhibitors of PKC and ERK. RPE50 cells were cultivated with a wound healing culture insert for 24 h. Cells were then untreated (-), treated with vitreous fluids PVR-vitreo, PDR-vitreo and RD-vitreo obtained from patients of PVR, PDR and RD, respectively, for 18 h. Each vitreous fluid was normalized with equal amount of protein (10–20 µg). Wound healing assay were performed and quantitated as described in **Figure 1 B**. The results were averages of 3 experiments with coefficient of variation of 7.5%. (*) and (**) represent statistical significance (P<0.05 and P<0.005, respectively) for comparison of the vitreous fluid treated-sample *vs.* untreated sample. In each vitreous fluid–treated group, (^#^) and (^##^) represent statistical significance (P<0.05 and P<0.005, respectively) for comparison of the inhibitor- *vs.* DMSO-treated group.

Finally, whether clinical samples from PVR and PDR patients may induce cell migration of RPE *via* the ERK/PKC signal pathway was investigated. Clinical vitreous fluids denoted as PVR-vitreo, PDR-vitreo and RD-vitreo were surgically obtained from patients with PVR, PDR and retinal detachment (RD), respectively. Both PVR-vitreo and PDR-vitreo samples contained abnormally higher HGF (1980 and 3800 pg/ml, respectively) as compared with that in RD-vitreo sample (127 pg/ml). However, both EGF and HB-EGF were not detected in these samples. Wound healing assay using the vitreous fluids (20 µg protein each sample) demonstrated that PVR-Vitro and PDR-Vitro greatly elevated motility of RPE50 by 2.3 and 4.3-fold, respectively, as compared with basal motility of untreated cells, whereas RD-vitro increased cell motility by only 0.8-fold (**Figure** 7). Interestingly, BIS, Rottlerin or PD98059 (but not DMSO) dramatically suppressed cell migration of RPE50 induced by all the vitreous fluids. This result indicates that activation of PKCδ and ERK are required for cell migration of RPE induced by HGF-containing vitreous fluids.

## Discussion

In this report, we demonstrated that cell migration of RPE cells was synergistically induced by HGF coupled with HB-EGF or EGF. All these factors were found to be elevated in PVR retinas, epiretinal membranes and vitreous, playing essential role in PVR process [7], [8]. Previous studies demonstrated that ERK (MAPK) was the common down stream signal kinase required for cell migration of RPE. We further found that PKC acts upstream of ERK for cell migration of RPE induced by HGF, EGF and HB-EGF (**Figure 2**). This is consistent with the previous studies demonstrating the role of PKC-ERK cascade in mediating cell migration of a lot of tumor cells induced by metastatic factors including HGF [38].

The underlying mechanisms for synergistic effect of HGF and EGF (or HB-EGF) on activating both ERK and PKC and enhancing cell migration of RPE were not fully understood. In the previous study [17] HGF transactivated EGFR which might be responsible for signal cross talk triggered by HGF and HB-EGF, however, whether HB-EGF may also transactivate HGF/c-met was not certain. In our results, we found inhibitors of EGF and HGF receptors prevented RPE cell migration, PKC activation and ERK phosphorylation reciprocally induced by HGF and EGF (**Figure 6**). This suggested that both growth factor receptors may not only be activated by each own ligand but also cross-activated by heterogeneous ligand, which enhanced signal transduction and cell migration.

Our results suggested that PKCδ is the most important PKC isozyme in mediating cell migration of RPE induced by aforementioned growth factors. Previous studies demonstrated the role of PKCδ in cell migration induced by EGF and PDGF [39], [40]. Moreover, PKCδ was able to transactivate c-met and EGFR [41], [42]. Therefore, it is tempting to suggest that PKCδ plays an essential role in signal transduction mediating PVR-related pathological process. In the future, it is worthy to investigate whether PKCδ can serve as a more specific target for prevention of PVR using chemo- or gene therapy.

Finally, we tried to validate our results using vitreous samples from PVR and PDR patients. The reason why only HGF (but not EGF and HB-EGF) was detected in the vitreous fluids was not clear. Probably, the PVR-related growth factors appeared in the vitreous fluids in a tempo-spatial fashion, thus only some of them can be detected in a specific sample obtained at the time of surgery. Nevertheless, vitreous fluids with higher HGF obtained from PVR and PDR patients induced higher motility of RPE than did vitreous fluids with lower HGF obtained from RD patient (**Figure** 7). Moreover, such vitreous-induced migration of RPE can be prevented by BIS, PD98059 and Rottlerin. This result suggests that inflammatory growth factors elevated in the vitreous of PVR or PDR may indeed trigger PVR-related phenotypical changes of RPE via PKC/ERK signal pathway as delineated in this study.

## Supporting Information

Figure S1 **Growth factors induced cell migration of RPE50.** RPE 50 were cultivated with a wound healing culture insert and serum-starved for 48 h. After removal of culture insert (0 h), cells were treated with 50 nM of HGF (H50), EGF (E50), TGFβ1 (T50) or PDGF (P50), 50 ng/ml HB-EGF (HB-E50), 25 nM HGF coupled with 25 nM EGF (H25/E25) or 25 nM HGF coupled with 25 ng/ml EGF (H25/HB-E25) in serum free medium for 18 h and photographed. The cells migrated into the blanking area were those appeared between orange lines which were depicted according the borderlines between the blanking area and cell culture boundary pictured at 0 h. The results were representatives of four reproducible experiments.
(TIF)

Figure S2 **Prevention of HBEGF-induced cell migration of RPE50 by various inhibitors of PKC isozymes.** RPE 50 cells were untreated (-), treated with 50 ng/ml HB-EGF, or HB-EGF coupled with DMSO (as solvent control) or various inhibitors as indicated. Wound healing assay were performed and quantitated as described in Fig. 1 A and B. The results were averages of 3 experiments with coefficient of variation (C.V.) of 5.5%. (**) represent statistical significance (P<0.005) for comparison of the inhibitor- *vs* DMSO-treated group.
(TIF)

Figure S3 **Time course of HB-EGF-induced ERK phosphorylation.** RPE50 cells were untreated, treated with 50 nM of HB-HGF for the time indicated. Western blot of p-ERK were performed using ERK nonspecific bands on Ponceaus stained blot as internal control. The relative ratio of the intensity for p-ERK/ERK (indicated below each lane) was calculated taking the ratio of the untreated group as 1.0. The results were averages of 2 experiments with coefficient of variation (C.V.) of 6.0–7.0%.
(TIF)

Figure S4 **over-expression of PKCδ elevated EGF- induced cell migration and ERK phosphorylation.** RPE50 cells were untransfected (MOCK), transfected with pcDNA or PKCδ for 36 h followed by treatment with 50 nM EGF for 24 h (A) or 30 min (C). Qunantitative migration was performed using wound healing method (B). Western blot of PKCδ (A) and ERK phosphorylation (C) were performed using nonspecific bands on Ponceaus stained blot (A) or ERK (C) as internal control. The relative ratios of the intensity for PKCδ/nonspecific band (A) and p-ERK/ERK (C) were calculated, taking the ratio of the pcDNA (A) or MOCK (C) as 1.0. In (B), (*) and (**) represent statistical significance (P<0.05 and P<0.005, respectively) for comparison of the EGF-treated *vs* untreated group. The results were averages of 2 experiments with coefficient of variation (C.V.) of 6.0–7.0%.
(TIF)
