# Supplementary figures and images for: Enhanced PKCδ and ERK Signaling Mediate Cell Migration of Retinal Pigment Epithelial Cells Synergistically Induced by HGF and EGF
Source: PLoS One. 2012 Sep 20;7(9):e44937. doi: 10.1371/journal.pone.0044937 (PMC3447816; doi:10.1371/journal.pone.0044937)

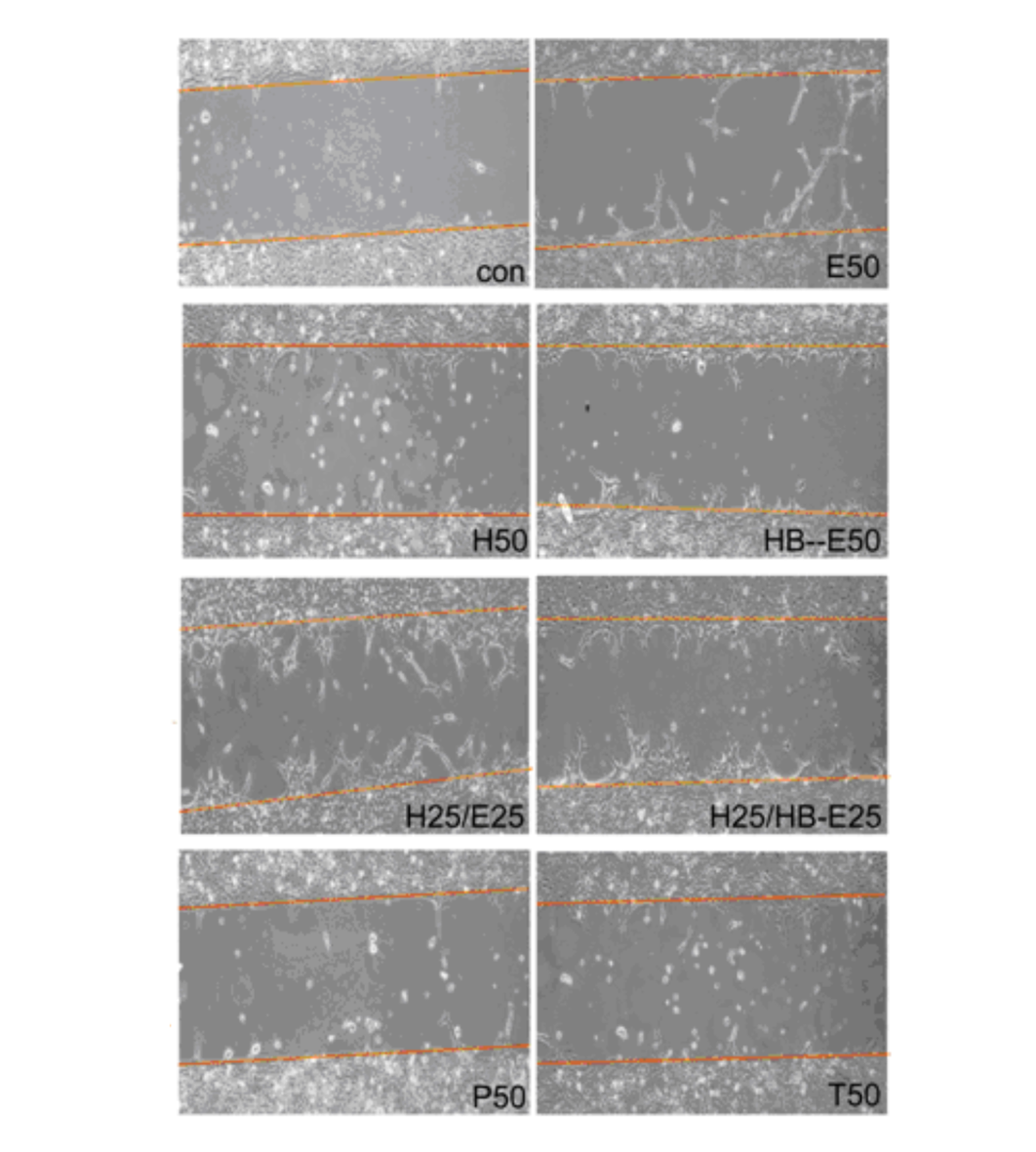

Supplement: Figure S1 — Growth factors induced cell migration of RPE50. RPE 50 were cultivated with a wound healing culture insert and serum-starved for 48 h. After removal of culture insert (0 h), cells were treated with 50 nM of HGF (H50), EGF (E50), TGFβ1 (T50) or PDGF (P50), 50 ng/ml HB-EGF (HB-E50), 25 nM HGF coupled with 25 nM EGF (H25/E25) or 25 nM HGF coupled with 25 ng/ml EGF (H25/HB-E25) in serum free medium for 18 h and photographed. The cells migrated into the blanking area were those appeared between orange lines which were depicted according the borderlines between the blanking area and cell culture boundary pictured at 0 h. The results were representatives of four reproducible experiments. (TIF) [file pone.0044937.s001.tif]

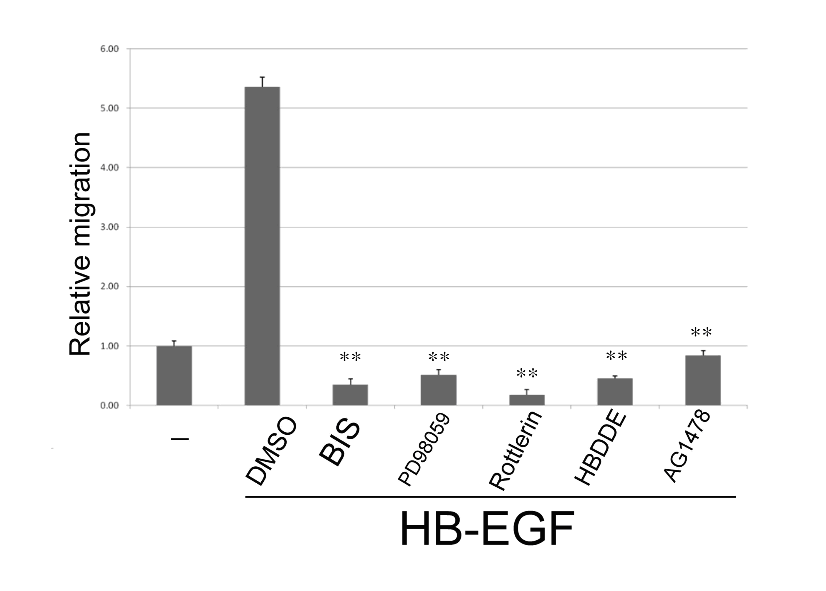

Supplement: Figure S2 — Prevention of HBEGF-induced cell migration of RPE50 by various inhibitors of PKC isozymes. RPE 50 cells were untreated (-), treated with 50 ng/ml HB-EGF, or HB-EGF coupled with DMSO (as solvent control) or various inhibitors as indicated. Wound healing assay were performed and quantitated as described in Fig. 1 A and B. The results were averages of 3 experiments with coefficient of variation (C.V.) of 5.5%. (**) represent statistical significance (P<0.005) for comparison of the inhibitor- vs DMSO-treated group. (TIF) [file pone.0044937.s002.tif]

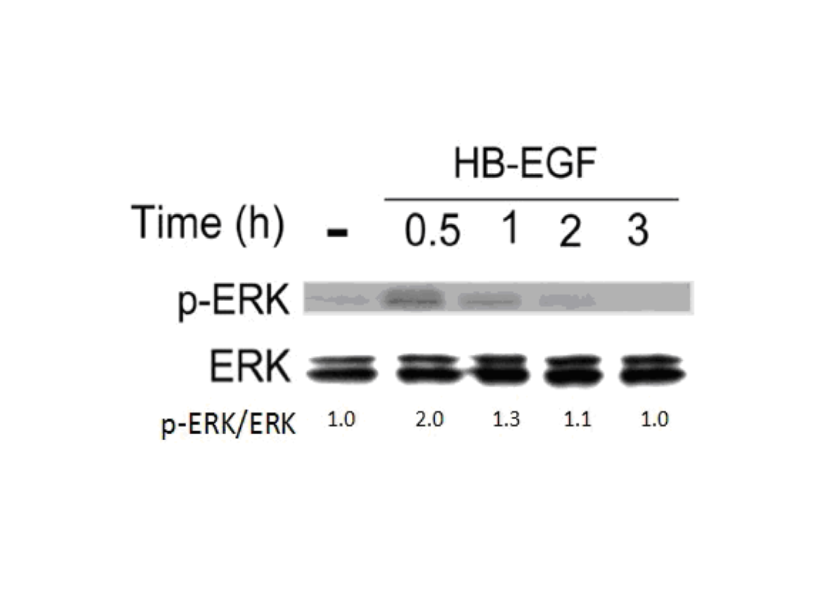

Supplement: Figure S3 — Time course of HB-EGF-induced ERK phosphorylation. RPE50 cells were untreated, treated with 50 nM of HB-HGF for the time indicated. Western blot of p-ERK were performed using ERK nonspecific bands on Ponceaus stained blot as internal control. The relative ratio of the intensity for p-ERK/ERK (indicated below each lane) was calculated taking the ratio of the untreated group as 1.0. The results were averages of 2 experiments with coefficient of variation (C.V.) of 6.0–7.0%. (TIF) [file pone.0044937.s003.tif]

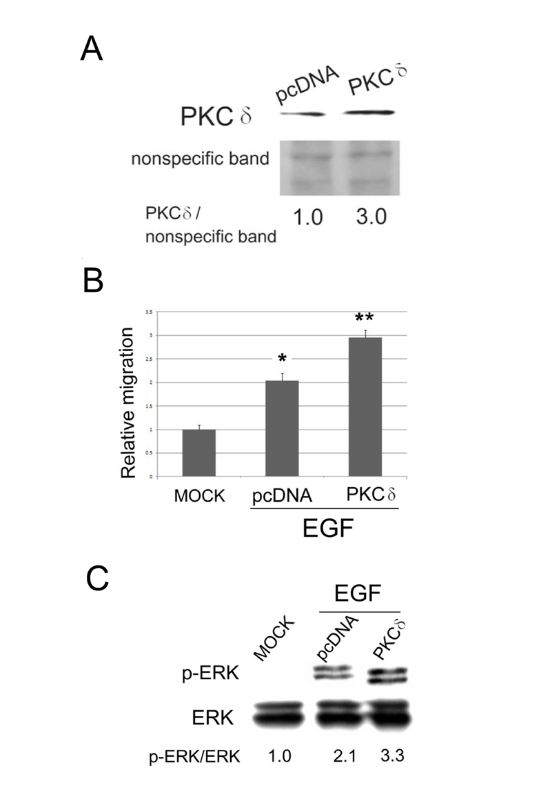

Supplement: Figure S4 — over-expression of PKCδ elevated EGF- induced cell migration and ERK phosphorylation. RPE50 cells were untransfected (MOCK), transfected with pcDNA or PKCδ for 36 h followed by treatment with 50 nM EGF for 24 h (A) or 30 min (C). Qunantitative migration was performed using wound healing method (B). Western blot of PKCδ (A) and ERK phosphorylation (C) were performed using nonspecific bands on Ponceaus stained blot (A) or ERK (C) as internal control. The relative ratios of the intensity for PKCδ/nonspecific band (A) and p-ERK/ERK (C) were calculated, taking the ratio of the pcDNA (A) or MOCK (C) as 1.0. In (B), (*) and (**) represent statistical significance (P<0.05 and P<0.005, respectively) for comparison of the EGF-treated vs untreated group. The results were averages of 2 experiments with coefficient of variation (C.V.) of 6.0–7.0%. (TIF) [file pone.0044937.s004.tif]
